# Human Infections by Novel Zoonotic Species *Corynebacterium silvaticum*, Germany

**DOI:** 10.3201/eid3107.250086

**Published:** 2025-07

**Authors:** Anja Berger, Alexandra Dangel, Vyacheslav G. Melnikov, Katja Bengs, Thomas Rupp, Hanns-Joerg Mappes, Christian Schneider, Andreas Sing

**Affiliations:** Bavarian Health and Food Safety Authority, Oberschleißheim, Germany (A. Berger, A. Dangel, K. Bengs, A. Sing); German National Consiliary Laboratory for Diphtheria, Oberschleißheim (A. Berger, V.C. Melnikov, A. Sing); European Union Public Health Reference Laboratory for Diphtheria and Pertussis, Oberschleißheim (A. Berger, A. Sing); World Health Organization Collaborating Centre for Diphtheria, Oberschleißheim (A. Berger, A. Sing); Klinikum Aschaffenburg-Alzenau, Chirurgische Klinik, Aschaffenburg, Germany (T. Rupp, H.-J. Mappes); Klinikum Aschaffenburg-Alzenau, Zentrallabor, Germany (C. Schneider)

**Keywords:** *Corynebacterium silvaticum*, bacteria, zoonoses, diphtheria, zoonotic, lymphadenitis, Germany

## Abstract

We report 2 human *Corynebacterium silvaticum* infections in Germany with axillary lymphadenitis and abscess formation; in 1 case the infection likely originated from a slaughtered wild boar. This recently described member of the diphtheria toxin gene–bearing *C. diphtheria*e species complex might be a new zoonotic pathogen.

Diphtheria is caused by toxigenic *Corynebacterium* spp. producing the major pathogenicity factor diphtheria toxin (DT). DT is encoded on the *tox* gene and causes local respiratory or cutaneous symptoms, as well as systemic neurologic and cardiologic symptoms. The 3 potentially DT-producing species are the mainly human pathogen *C. diphtheriae* and the 2 zoonotic pathogens *C. ulcerans* and *C. pseudotuberculosis*. In recent years, the *C. diphtheriae* species complex (CdSC) was expanded, mostly on the basis of genomic data, in some instances supported by biochemical properties (*1*). Two closely related species were separated from *C. diphtheriae* (i.e., *C. belfantii* [*2*] and *C. rouxii* [*3*]), and 2 were separated from *C. ulcerans* (i.e., *C. silvaticum* [*4*] and *C. ramonii* [*5*], previously known as lineage 2 of *C. ulcerans*).

*C. silvaticum* (formerly *C. ulcerans* wildlife cluster), was recently described as a novel zoonotic species causing caseous lymphadenitis in 33 wild boars and a roe deer in Germany (*4*,*6*). Subsequently, *C. silvaticum* was identified in a wild boar from Germany (*7*) and in a domestic pig from Portugal (*8*). So far, all animal isolates from Germany were nontoxigenic *tox*-bearing (NTTB) (*4*,*8*,*9*), whereas isolates from Portugal and Austria were found to be either NTTB or *tox*-positive, resulting in 2 recently postulated clades depending on molecular characteristics of the *tox* gene (*10*). Although *C. silvaticum* is considered a zoonotic pathogen with the potential to infect humans, no human infections had been described. In this study, we report 2 cases of human *C. silvaticum* infection. 

## The Study

In case 1, a 37-year-old male butcher sought care for an indolent tumor in the lateral thorax and the right axilla that had been present for 3 weeks. A bacterial swab specimen taken during surgical extirpation grew *Corynebacterium* sp. after 48 hours in pure culture; the pathogen was *C. ulcerans* by matrix-assisted laser desorption/ionization time-of-flight mass spectrometry (Bruker, https://www.bruker.com). The strain was sent to the German Consiliary Laboratory for Diphtheria (Oberschleißheim, Germany) for further analysis. The strain (KL1848) showed an atypical colony morphology (small waxy white, discrete β-hemolytic colonies after 48 hours’ incubation) and biochemical reactions: fermentation of glucose, ribose, and maltose (like *C. ulcerans*), but no use of D-xylose, mannitol, lactose, sucrose, or glycogen (like *C. pseudotuberculosis*). It was sensitive to clindamycin, according to the European Committee on Antimicrobial Susceptibility Testing guidelines for *C. diphtheriae* and *C. ulcerans* (https://www.eucast.org/clinical_breakpoints). The isolate was identified as a NTTB *C. silvaticum* strain using previously described methods (*11*) (i.e., *tox*-PCR, an optimized Elek test, a lateral flow immunoassay and whole-genome sequencing [WGS] analyses) (Appendix 1). The patient was successfully treated with surgery and cefuroxime for 14 days. The patient reported to have recently field-dressed a hunted wild boar that showed suspicious mesenterial lymph nodes. The patient’s immunization status was unknown. No secondary human cases occurred.

In case 2, a 21-year-old man sought care for an indolent axillary tumor that had been present for 4 weeks. Lymphoma was suspected on the basis of computed tomography results. The affected lymph node was removed, revealing a fibrosed soft tissue with florid purulent abscess formation and chronic granulating histiocyte-rich granulomatous inflammation. After a few days of incubation, the tissue sample grew a pure culture of *Corynebacterium* sp., identified as *C. ulcerans* by matrix-assisted laser desorption/ionization time-of-flight mass spectrometry (Bruker). The strain (KL1281) was sent to the German Consiliary Laboratory for Diphtheria. The atypical colony morphology, biochemical reactions, and antimicrobial susceptibility were identical to those of strain KL1848. We identified the isolate as NTTB *C. silvaticum* using the same methods as in case 1. The case 2 patient had a dog and lived in a rural area of northern Germany. He reported no contact with wild animals. Vaccination against diphtheria was completed. No secondary human case was observed.

Average nucleotide identity analysis of WGS assemblies compared with public type-strain genomes of the CdSC classified both isolates with highest concordance as *C. silvaticum* with identity values >99.9%. Identity values for the other species were nearly identical for both strains and clearly below the species threshold of 94%–96% (*12*); values were 90.6% for *C. ramonii* FRC0011^T^, 90.5% for *C. ulcerans* NCTC7910^T^, 85.0% for *C. pseudotuberculosis* ATCC19410^T^, 74.3% for *C. diphtheriae* NCTC11397^T^, 74.1% for *C. rouxii* FRC190^T^, and 74.0% for *C. belfantii* FRC0043^T^.

**Figure 1:**
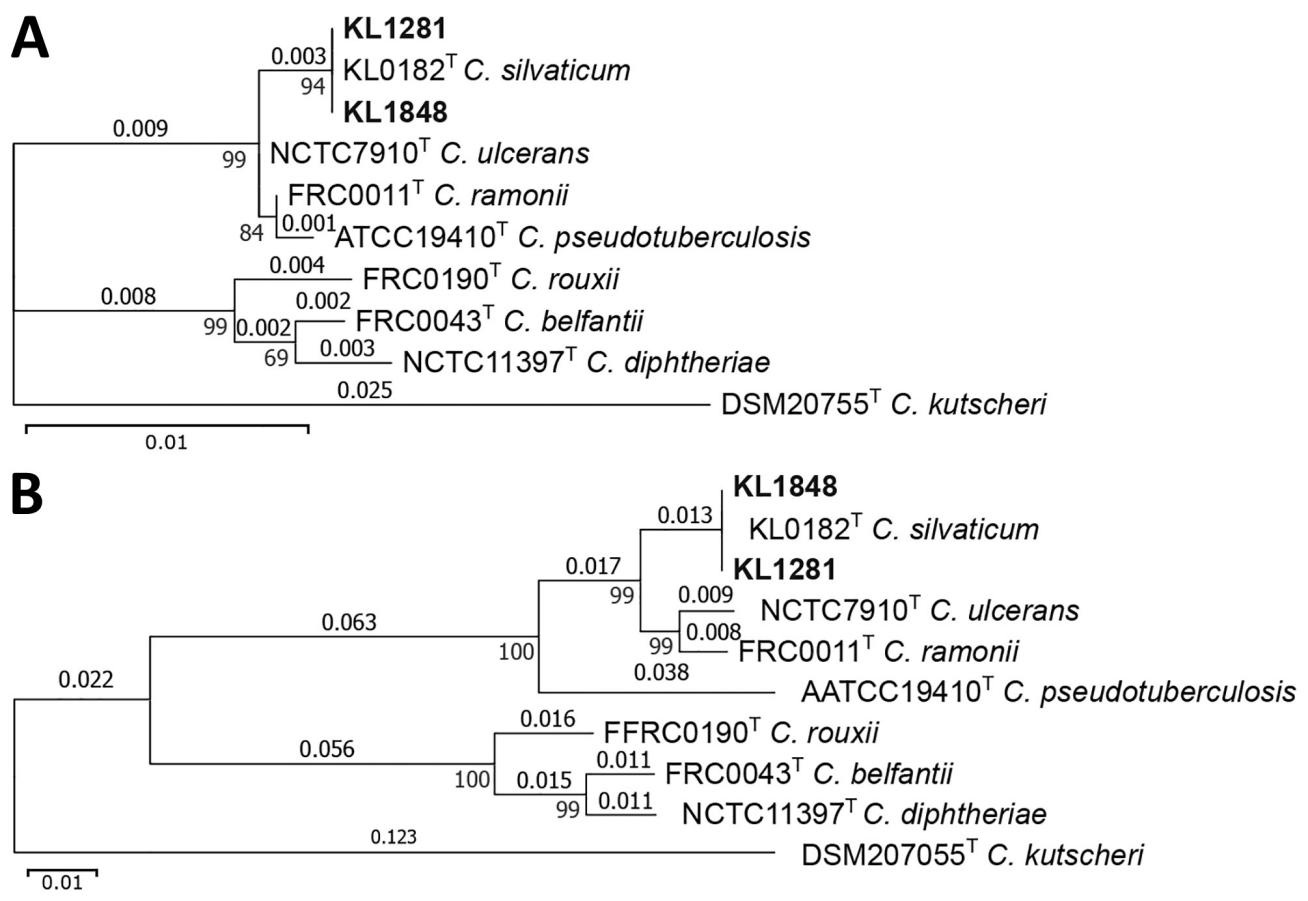
Gene phylogenies of isolates from 2 cases of infection by the novel zoonotic species *Corynebacterium silvaticum*, Germany (isolates KL1281 and KL1848), compared with reference sequences from publicly available type strain sequences of *C. diphtheriae* complex species and a close relative outgroup (*C. kutscheri*)*.* A) 16S; B) rpoB. Substitutions per aligned site are indicated above branches and local supporting values at intersections of branches. Scale bars indicate number of substitutions per site.

We constructed gene phylogenies of 16S and *rpoB* from the WGS data and compared them with the respective genes of the CdSC-type strains and *C. kutscheri* as an outgroup. The phylogenetic trees (Figure 1) show a clear assignment to *C. silvaticum*, because both isolates share the branch of the *C. silvaticum*–type strain with clear separation from the other species. Multilocus sequence typing (MLST) analysis with the *C. diphtheriae* scheme showed sequence type (ST) 578, exclusive to *C. silvaticum* and typically also found in isolates from wild boar and roe deer in Germany (Appendix 2). Evaluation of the *tox* gene and an alignment with *tox* sequences from Germany, published sequences from Portugal (*10*) and a *C. diphtheriae tox* sequence as reference revealed the presence of the insertion of 2 guanine residues after position 44 (Appendix 1 Figure), as found in the other Germany isolates from wild boar and roe deer, enabling assignment to *C. silvaticum* clade 2 (*10*).

**Figure 2:**
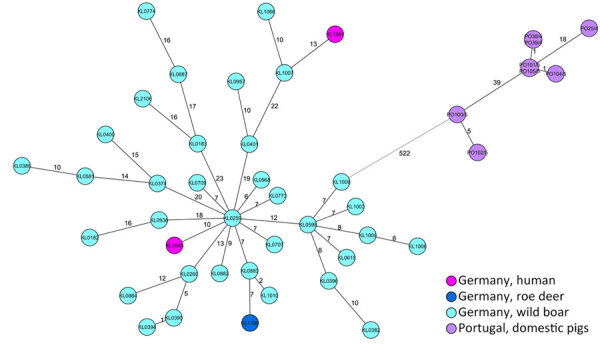
Minimum-spanning tree of an ad hoc *Corynebacterium silvaticum* core-genome multilocus sequence typing scheme of 2012 target loci displaying isolates from 2 cases in Germany (KL1281 and KL1848), and reference sequences. Shown are relationships with 35 animal-derived isolates from Germany and 8 isolates from clade 1 from pigs in Portugal. Allelic distances are indicated at connection lines.

To investigate the clade classification with a more detailed core genome MLST analysis, we constructed an ad hoc core genome MLST scheme on the basis of publicly available *C. silvaticum* genomes from both clades (Appendix 1). We generated a minimum-spanning tree from 45 genomes, consisting of KL1281, KL1848, an additional 35 isolates from animals from Germany, and the 8 clade 1 genomes from Portugal (*10*). The tree layout (Figure 2) and the allelic distances (Appendix 2) clearly affiliate the patient cases with the cases from clade 2 from Germany, whereas the genomes from clade 1 from Portugal sit on another branch and show >500 allelic differences to clade 2. Thus, the classification by the *tox* gene is also supported by a whole-genome context.

## Conclusions

*C. silvaticum* (*4*) has been suspected to be a possible zoonotic member of the newly expanded CdSC. Originally isolated from forest-associated animals such as wild boars and roe deer, it has also been identified in a domestic pig. In this study, we report 2 cases of human infection linked to close animal contact (wild boar and dog). In case 1, the most probable route of infection was direct contact with infectious tissue and possible microtrauma lesions during a slaughtering or animal field-dressing procedure. In case 2, no close contact to wild animals was reported, although the patient lived in a rural area and owned a dog. However, *C. silvaticum* has so far been only identified in wild forest-dwelling animals and a pig. All cases in Germany, including the 2 human cases described in this study, were found to be NTTB and summarized in a corresponding NTTB clade with a 2-bp insertion in the *tox* gene sequence, named clade 2 (*10*). In contrast, in Portugal and Austria, another monophyletic clade of DT-producing *C. silvaticum* was reported and named clade 1 (*10*).

In conclusion, *C. silvaticum* should be considered a zoonotic pathogen with possible animal-to-human transmission. Therefore, animal sources, especially wild boar and domestic pigs, should be included when tracing sources of potentially toxigenic *Corynebacterium* spp. of the broader *C. diphtheriae* and the narrower *C. ulcerans* complex. *C. ulcerans* has also been transmitted from pigs to humans (*13*,*14*). With respect to the known clindamycin resistance in *C. ulcerans* (*15*), antimicrobial susceptibility testing should support antibiotic therapy. Suitable precaution measures should be taken when handling wild boars and pigs to avoid zoonotic risk for *C. ulcerans* or *C. silvaticum* infection.

Appendix 1 Additional information about human infections by the novel zoonotic species *Corynebacterium silvaticum*, Germany.

Appendix 2 Additional data from study about human infections by the novel zoonotic species *Corynebacterium silvaticum*, Germany.
