# Supplementary material for: Human Infections by Novel Zoonotic Species Corynebacterium silvaticum, Germany
Source: Emerg Infect Dis. 2025 Jul;31(7):1450–4. doi: 10.3201/eid3107.250086 (PMC12205466; doi:10.3201/eid3107.250086)
Supplement: Appendix 1 — Additional information about human infections by the novel zoonotic species Corynebacterium silvaticum, Germany. [file 25-0086-Techapp-s1.pdf]

Article DOI: https://doi.org/10.3201/eid3107.250086

EID cannot ensure accessibility for supplementary materials supplied by authors. Readers who have difficulty accessing supplementary content should contact the authors for assistance.

Human Infections by the Novel Zoonotic Species *Corynebacterium silvaticum*, Germany

Appendix

Supplemental Material and Methods for WGS analyses

Whole genome sequencing (WGS) wetlab analyses

WGS analyses were performed starting with extraction of genomic DNA from overnight cultures in Brain-Heart-Infusion broth, pelleted and resuspended in 50 µl PBS, using the GenFind V3 Genomic DNA Extraction Kit on a Biomek i7 machine (both Beckman Coulter, Indianapolis, IN, USA) with preceding incubation with 7 mg/µl Lyzozyme for 30h at 37°C. WGS library preparation and sequencing was performed using the Illumina DNA prep kit and paired-end sequencing on Illumina MiSeq or NextSeq550 sequencers with 2x150 or 2x250 paired-end reads.

WGS data analyses

WGS data analyses were generally performed as described in (*1*).

After raw data quality control with fastQC v0.11.9 (*2*) and kraken2 v2.1.2 (*3*), fastq raw data were quality-trimmed with trimmomatic v0.39 (*4*). WGS assemblies were generated using SPAdes v3.15.5 (*5*) and assemblies were again checked for quality using quast v5.3.0 (*6*) and annotated with prokka v1.14.6 (*7*).

For *16S* and *rpoB* phylogenies and *tox* gene alignments sequences of the respective genes were extracted from the annotated WGS assemblies. For publicly available type strain genomes of the CdSC and *C. kutscheri* as an outgroup-species, closely related to but outside the CdSC,

genes were likewise extracted or downloaded from NCBI (accession numbers and gene lengths for public of *16S* and *rpoB* from publicly available genomes of type strains: *C. silvaticum* NCTC7910^T: *16S*: MK602323 (1518 nt), *rpoB*: GCA_004382825.2 (3537 nt), *C. ulcerans* NCTC7910^T: NZ_LT906443.1 (*16S*: 1528 nt, *rpoB*: 3537nt), *C. diphtheriae* NCTC11397^T: NZ_LN831026.1 (*16S*: 1527 nt, *rpoB*: 3495 nt), *C. pseudotuberculosis* ATCC19410^T: GCF002155265.1 (1528 nt, *rpoB*: 3501 nt), *C. rouxii* FRC0190^T: *16S*: MN535983 (1533 nt), *rpoB*: MN542348 (3531 nt), *C. belfantii* FRC0043^T: 16S: LT960553.1 (1530 nt), *rpoB*: GCA_900205605.1 (3531 nt), *C. ramonii* FRC0011^T: OX444692.1 (16S: 1517 nt, *rpoB*: 3537 nt), *C. kutscheri* DSM20755^T: CP011312.1 (*16S*: 1528 nt, *rpoB*: 3477 nt).

Gene sequences were aligned with muscle v5.1 (*8*) and approximately-maximum-likelihood trees were built for *16S* and *rpoB* using fasttree 2.1 (*9*).

ANI analysis was carried out with the genome assemblies using PyANI v0.2.12 (*10*), based on blastn+ algorithm using the species boundary concept of (*11*) and (*12*).

MLST and cgMLST was done with the WGS assemblies using Ridom SeqSphere+ v10.0 (Ridom, Münster, Germany). For MLST seven housekeeping loci *atpA*, *dnaE*, *dnaK*, *fusA*, *leuA*, *odhA*, *rpoB* of the *C. diphtheriae* scheme were analyzed (*13*,*14*) and matched to public MLST database (https://bigsdb.pasteur.fr/diphtheria/). For cgMLST an ad hoc scheme for *C. silvaticum* with 2012 target loci, generated as describes below, was used for typing.

Generation of an ad hoc cgMLST scheme

An ad hoc cgMLST scheme was constructed in Ridom SeqSphere+ software v10.0 (Ridom GmbH, Munster, Germany), based on publicly available *C. silvaticum* genomes from both Clades postulated by (*15*). Therefore, public genomes with high completeness were used, including type strain KL0182^T and KL0957 from wild boar, as well as KL1196 from roe deer from Germany, Austrian wild boar genomes and Portuguese genomes from domestic pigs. The scheme resulted in 2012 *C. silvaticum* specific core genome target loci.

The following settings were used for generation of the scheme:

Seed Genome: NZ_CP081180.1 (06-NOV-2024), 2572991 bases, 2374 genes with CDS (*C. silvaticum* strain PO101/5 chromosome, complete genome). Penetration Query Genomes (*11*): NZ_CP080459.1 (20-SEP-2024), 2572936 bases, 2388 genes with CDS (*C. silvaticum*

strain PO102/5 chromosome, complete genome); NZ_CP080461.1 (20-SEP-2024), 2572825 bases, 2372 genes with CDS (*C. silvaticum* strain PO25/4 chromosome, complete genome); NZ_CP081181.1 (06-NOV-2024), 2572843 bases, 2386 genes with CDS (*C. silvaticum* strain PO105/5 chromosome, complete genome); NZ_CP081182.1 (06-NOV-2024), 2572864 bases, 2387 genes with CDS (*C. silvaticum* strain PO38/4 chromosome, complete genome); NZ_CP081179.1 (06-NOV-2024), 2572860 bases, 2384 genes with CDS (*C. silvaticum* strain PO39/4 chromosome, complete genome); SDQO00000000.2, 2554413 bases, 13 contigs (*C. silvaticum* strain KL0182, whole genome shotgun sequencing project.); SDVC00000000.1, 2554065 bases, 27 contigs (*C. silvaticum* strain KL0957, whole genome shotgun sequencing project.); SDVD00000000.1, 2550347 bases, 20 contigs (*C. silvaticum* strain KL1196, whole genome shotgun sequencing project.); VFEM00000000.1, 2550924 bases, 13 contigs (*C. silvaticum* strain W25, whole genome shotgun sequencing project.); JABGCN000000000.1, 2548286 bases, 12 contigs (*C. silvaticum* strain 04–13, whole genome shotgun sequencing project.); JABGCO000000000.1, 2551141 bases, 18 contigs (*C. silvaticum* strain 05–13, whole genome shotgun sequencing project.); JAEANX000000000.1, 2550121 bases, 8 contigs (*C. silvaticum* strain 5182, whole genome shotgun sequencing project.)

Seed Genome Filters: Minimum Length Filter (requires > = 50 bases); Start Codon Filter (requires start codon at beginning of the gene); Stop Codon Filter (requires single stop codon at end of gene); Homologous Gene Filter (requires no multiple copies of gene with BLAST overlap> = 100bp, identity> = 90.0%); Gene Overlap Filter (requires no overlap with other genes >4 bases)

Query Genome BLAST Search: Requires BLAST hit with overlap = 100%, identity> = 90.0% in every query genome; BLAST options: Word size = 11, Mismatch penalty = −1, Match reward = 1, Gap open costs = 5, Gap extension costs = 2; Penetration Query Genomes Filters: Stop Codon Percentage Filter (requires single stop codon at end of gene in >80% penetration query genomes); BLAST version 2.2.12.

References

1. Dangel A, Berger A, Rau J, Eisenberg T, Kämpfer P, Margos G, et al. *Corynebacterium silvaticum* sp. nov., a unique group of NTTB corynebacteria in wild boar and roe deer. Int J Syst Evol Microbiol. 2020;70:3614–24. PubMed https://doi.org/10.1099/ijsem.0.004195

2. Andrews S. FastQC: a quality control tool for high throughput sequence data. http://www. bioinformatics.babraham.ac.uk/projects/fastqc 2010.

3. Wood DE, Salzberg SL. Kraken: ultrafast metagenomic sequence classification using exact alignments. Genome Biol. 2014;15:R46. PubMed https://doi.org/10.1186/gb-2014-15-3-r46

4. Bolger AM, Lohse M, Usadel B. Trimmomatic: a flexible trimmer for Illumina sequence data. Bioinformatics. 2014;30:2114–20. PubMed https://doi.org/10.1093/bioinformatics/btu170

5. Bankevich A, Nurk S, Antipov D, Gurevich AA, Dvorkin M, Kulikov AS, et al. SPAdes: a new genome assembly algorithm and its applications to single-cell sequencing. J Comput Biol. 2012;19:455–77. PubMed https://doi.org/10.1089/cmb.2012.0021

6. Gurevich A, Saveliev V, Vyahhi N, Tesler G. QUAST: quality assessment tool for genome assemblies. Bioinformatics. 2013;29:1072–5. PubMed https://doi.org/10.1093/bioinformatics/btt086

7. Seemann T. Prokka: rapid prokaryotic genome annotation. Bioinformatics. 2014;30:2068–9. 10.1093/bioinformatics/btu153 PubMed https://doi.org/10.1093/bioinformatics/btu153

8. Edgar RC. MUSCLE: a multiple sequence alignment method with reduced time and space complexity. BMC Bioinformatics. 2004;5:113. PubMed https://doi.org/10.1186/1471-2105-5-113

9. Price MN, Dehal PS, Arkin AP. FastTree 2—approximately maximum-likelihood trees for large alignments. PLoS One. 2010;5:e9490. 10.1371/journal.pone.0009490 PubMed https://doi.org/10.1371/journal.pone.0009490

10. Pritchard L. PyANI. Copyright The James Hutton Institute 2014–2019. Pyani. Python module for Average Nucleotide Identity analyses [cited 2018]. https://github. com/widdowquinn/pyani

11. Richter M, Rosselló-Móra R. Shifting the genomic gold standard for the prokaryotic species definition. Proc Natl Acad Sci U S A. 2009;106:19126–31. PubMed https://doi.org/10.1073/pnas.0906412106

12. Goris J, Konstantinidis KT, Klappenbach JA, Coenye T, Vandamme P, Tiedje JM. DNA-DNA hybridization values and their relationship to whole-genome sequence similarities. Int J Syst Evol Microbiol. 2007;57:81–91. 10.1099/ijs.0.64483-0 PubMed https://doi.org/10.1099/ijs.0.64483-0

13. Bolt F, Cassiday P, Tondella ML, Dezoysa A, Efstratiou A, Sing A, et al. Multilocus sequence typing identifies evidence for recombination and two distinct lineages of *Corynebacterium diphtheriae.* J Clin Microbiol. 2010;48:4177–85. PubMed https://doi.org/10.1128/JCM.00274-10

14. König C, Meinel DM, Margos G, Konrad R, Sing A. Multilocus sequence typing of *Corynebacterium ulcerans* provides evidence for zoonotic transmission and for increased prevalence of certain sequence types among toxigenic strains. J Clin Microbiol. 2014;52:4318–24. PubMed https://doi.org/10.1128/JCM.02291-14

15. Viana MVC, Galdino JH, Profeta R, Oliveira M, Tavares L, de Castro Soares S, et al. Analysis of *Corynebacterium silvaticum* genomes from Portugal reveals a single cluster and a clade suggested to produce diphtheria toxin. PeerJ. 2023;11:e14895. PubMed https://doi.org/10.7717/peerj.14895

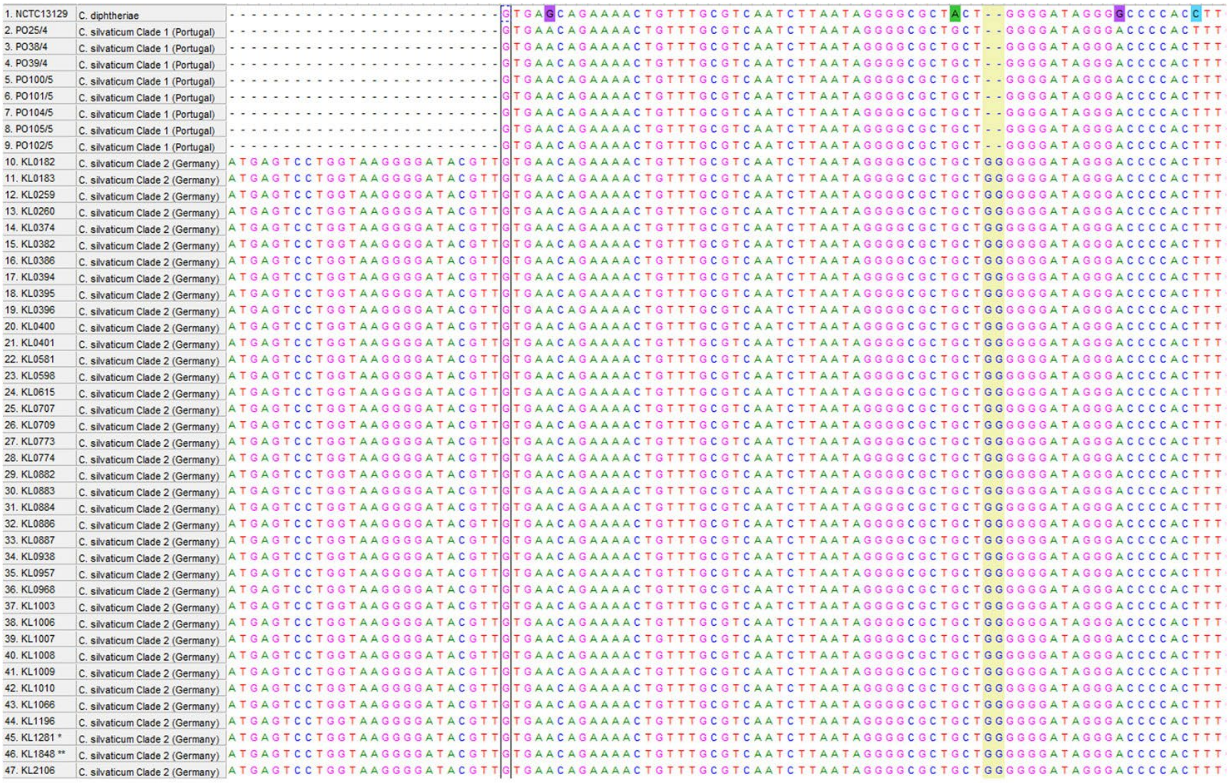

Appendix Figure. Excerpt from a *tox* gene alignment of *C. silvaticum* KL1281 and KL1848, together with the animal-derived *C. silvaticum tox* gene sequences from Germany (Clade 2, n = 35) and Portugal (Clade 1, n = 8, Viana et al. 2023) and *C. diphtheriae* (NCTC13129) *tox* gene as a reference. KL1281 is marked with an asterisk and KL1848 with two asterisks. Standard *tox* gene start is marked with a frame and the *C. silvaticum* Clade 2-specific insertion after base 44 from the gene start is marked with light yellow color. Bases differing between *C. diphtheriae* and *C. silvaticum* are also highlighted by base specific background colors (A = green, G = purple, C = blue). Accession numbers of the WGS data, from which the *tox* genes were extracted are given in Appendix 2 (https://wwwnc.cdc.gov/EID/article/31/7/25-0086-App2.xlsx).
